# The Microsurgical Translaminar Approach: A Technical Note

**DOI:** 10.7759/cureus.54334

**Published:** 2024-02-16

**Authors:** Asen H Cekov, Donika I Vezirska

**Affiliations:** 1 Department of Neurosurgery, Acibadem City Clinic University Hospital Tokuda, Sofia, BGR

**Keywords:** low back pain, lumbar disc herniation, minimally invasive lumbar approach, tissue-sparing technique, translaminar approach

## Abstract

Lumbar disc herniation (LDH) is a very common cause of low back pain and unilateral radiculopathy. This pathology is traditionally approached via interlaminotomy with variable amounts of bony extensions, such as partial or total facetectomy and disturbance of soft tissue structures. However, the extensive bone removal may be the cause of spinal instability, thus necessitating lumbar fusion procedures in the longer-term perspective. A novel surgical technique that spares the facet joint, the ligamentum flavum, and the epidural soft tissues was previously proposed. We present a detailed overview of the microsurgical translaminar approach for large LDH.

## Introduction

Lumbar disc herniation (LDH) are traditionally approached via interlaminotomy with variable amounts of bony extensions, such as partial or total facetectomy, and disturbance of the soft tissue structures, such as the ligamentum flavum and periradicular fat. However, the extensive bone removal may cause segmental instability and further lead to the need for lumbar fusion procedures. In 1998, Di Lorenzo et al. proposed a novel surgical technique that preserves the facet joint, the ligamentum flavum, and the epidural soft tissues [1]. This approach was met with skepticism regarding the technical difficulty, the risk of pars interarticularis fracture, and the supposed inability to execute a microdiscectomy, as well as the poor visual control of the nerve root and the epidural bleeding [2]. Further studies show that this approach is gaining new momentum as a method to access cranially and laterally migrated LDH and the lumbar hidden zone [3]. We demonstrate the feasibility of the microsurgical translaminar approach for large LDH.

## Technical report

Case illustration

**Figure 1:**
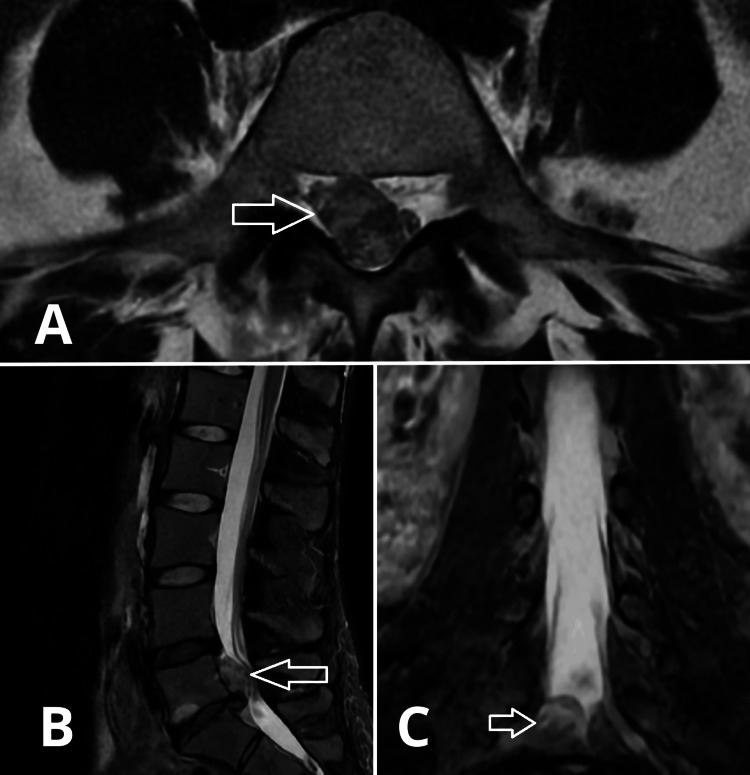
Pre-operative lumbar spine MRI scan revealed a large right-sided cranially migrated L5-S1 lumbar disc herniation (showed by a white arrow). A) Axial view. B) Sagittal view. C) Coronal view.

The patient is a 35-year-old woman with no previous history of low back pain who presented with right-sided L5 radiculopathy and right-sided peroneal paresis. MRI imaging revealed a large right-sided cranially migrated L5-S1 lumbar disc herniation (Fig. 1).

**Figure 2:**
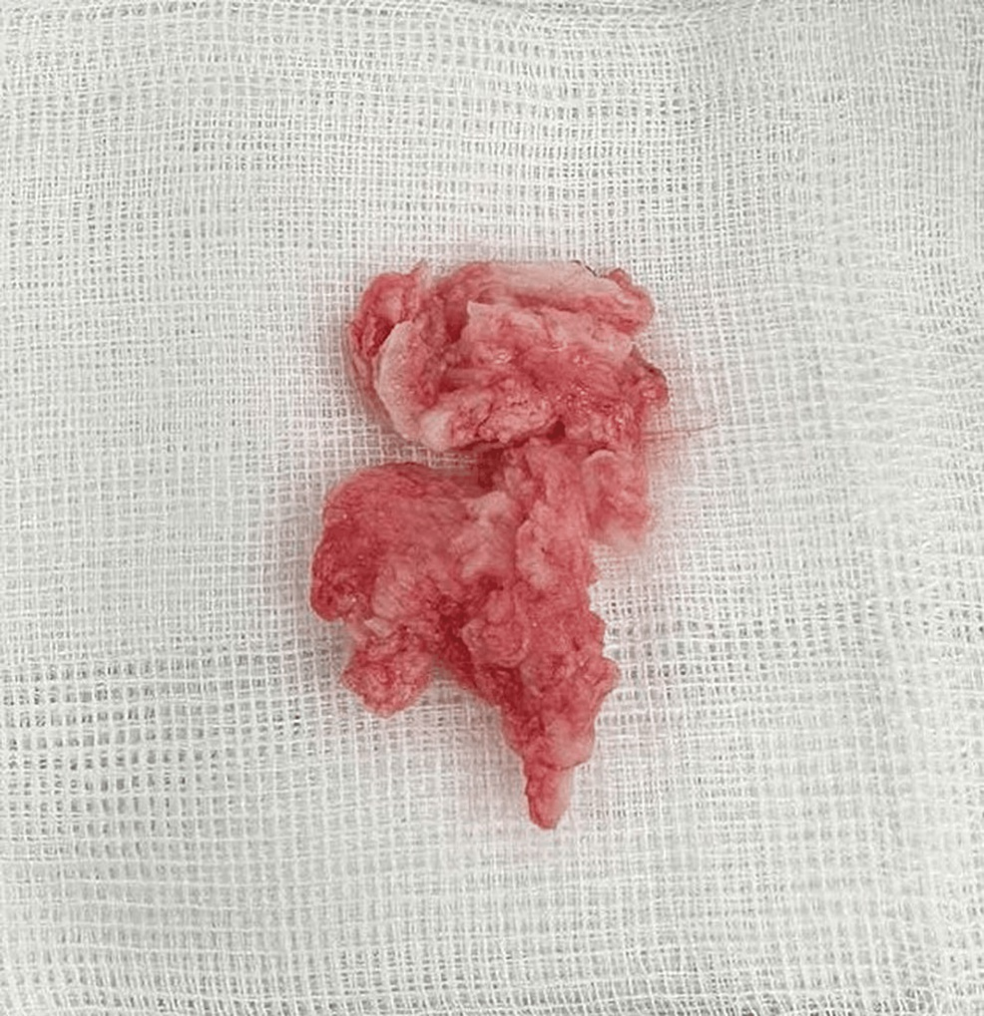
The removed herniated disc fragments were placed on a 7.5x7.5 cm white gauze.

She underwent a sequestrectomy via a right-sided microsurgical translaminar approach (Fig. 2).

**Figure 3:**
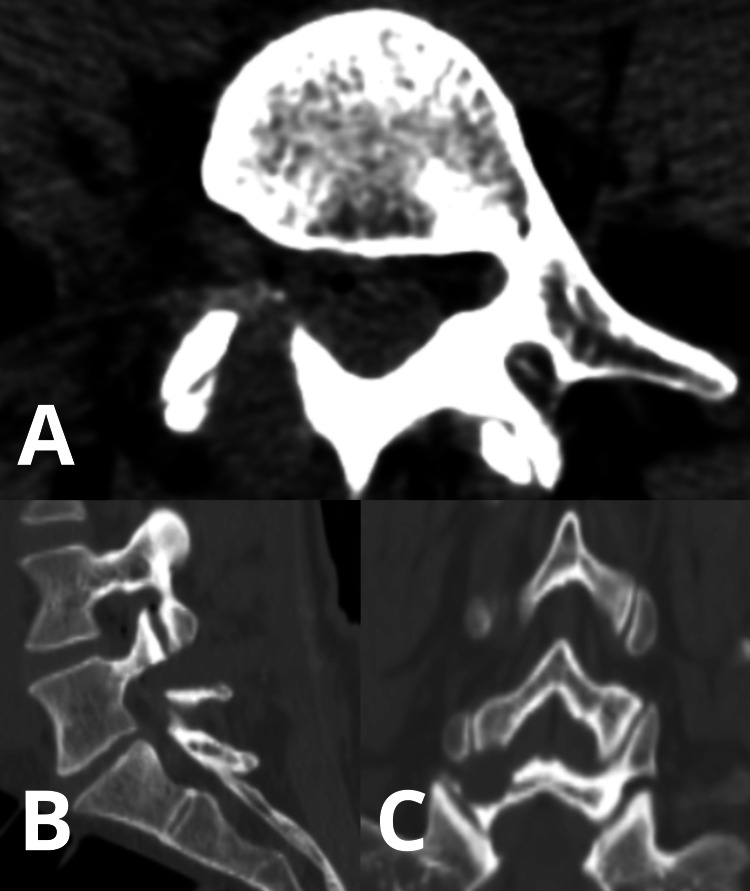
Post-operative lumbar spine CT scan showed no residual compression of the neural structures. The surgical corridor can be explored on the sagittal view. A) Axial view. B) Sagittal view. C) Coronal view.

She was mobilized later on the day of the surgery and reported a significantly lower VAS score (pre-operative 8.0 vs. post-operative 2.0). She was discharged on the first postoperative day without any neurological deficit. A lumbar CT scan was obtained on the day of the discharge - it showed that the herniated disc fragment was completely removed (Fig. 3).

At the one-month postoperative check-up examination, she was asymptomatic and had no residual neurological deficit.

Steps of the operative technique

After the induction of general anesthesia, the patient is placed prone on a monoblock bridge frame - it should be noted that the target lamina should be parallel to the floor for a more ergonomic position of the high-speed drill in a true perpendicular direction. The operating table is tilted in a head-upward direction to compensate for the otherwise oblique direction of the laminae. A localization X-ray image centered on the projected midpoint of the herniation is performed to verify the operative level.

**Figure 4:**
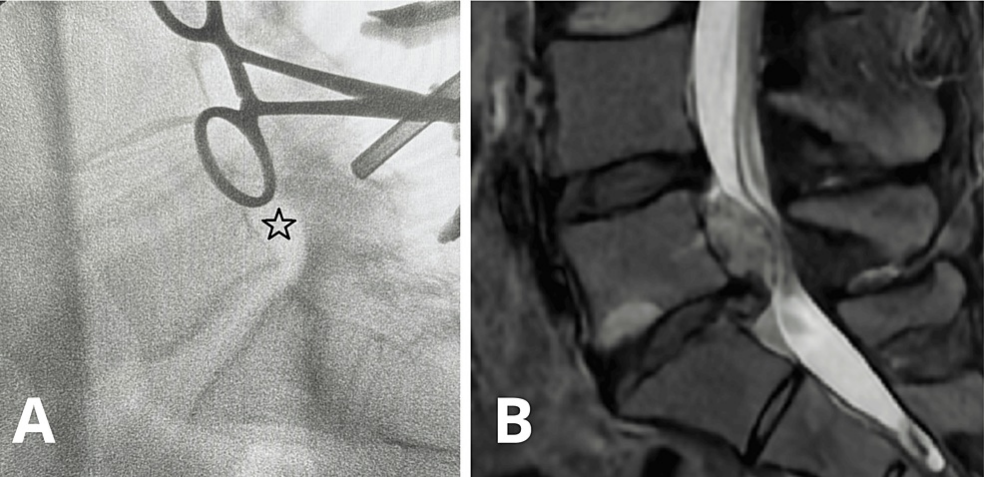
A) Intraoperative fluoroscopic image of L4-S1 levels showing the correspondence of the Caspar retractor system positioning to the preoperative MRI image (the projection of the herniated disc is marked with a black star sign). B) Sagittal view of the L5-S1 LDH on the preoperative MRI.

A 20-25 mm midline incision is performed, followed by a standard periosteal dissection of the paraspinal muscles unilaterally. The lamina and the ligamentum flavum are exposed. A Caspar-type retractor is then inserted. An intraoperative X-ray image is performed to verify the correct positioning of the retractor (Fig. 4).

High-speed drilling with a 6 mm cutting burr is used to form a round-shaped bone window in the lamina with an approximate diameter of 6-7 mm (Video 1).

**Video 1:** Drilling the lamina of the L5 vertebra with a 6 mm cutting burr.

**Figure 5:**
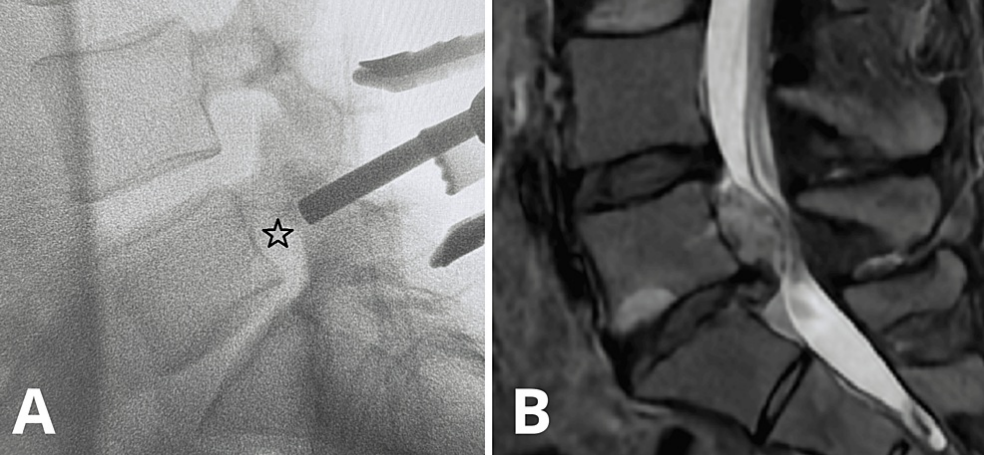
A) Intraoperative fluoroscopic image of L4-S1 levels showing the correspondence of the formed surgical corridor (marked with the aspiration tip) to the preoperative MRI image (the projection of the herniated disc is marked with a black star sign). B) Sagittal view of the L5-S1 LDH on the preoperative MRI.

An intraoperative X-ray image is performed to verify the correct trajectory and location of the operative corridor over the herniated disc fragment (Fig. 5).

The ligamentum flavum is released from the anterior part of the lamina with radial motions of the ball tip angled hook in a strongly perpendicular and subperiosteal fashion (Video 2).

**Video 2:** The release of the ligamentum flavum from the lamina.

Then, the ligamentum flavum is split parallel to the direction of the posterior radicular artery with the hook, and a 2 mm Kerrison punch is used to make a small hole in it for access to the herniation (Video 3).

**Video 3:** Splitting of the ligamentum flavum.

The herniated disc fragment is mobilized with different sizes of ball tip-angled hooks and dynamic retraction with the aspiration. It is then extracted with Caspar rongeurs, preferably in one whole piece (Video 4).

**Video 4:** Extraction of the disc fragment.

Epidural bleeding is managed with bipolar coagulation and, in more severe cases, hemostatic agents. After achieving hemostasis, standard layered closing is performed: the fascia is closed via continuous suture with Vicryl 1, the subcutaneous tissue - via continuous suture with Vicryl 2/0 and the skin is glued.

## Discussion

The microsurgical translaminar approach is an approach to the lumbar hidden zone and is a good option for patients with cranially and laterally migrated LDH - it is a good and cost-effective option for countries where endoscopic and/or exoscopic equipment is not widely available and used [2]. Vanni et al. suggested the use of this approach when MRI imaging shows a herniated fragment that is cranially migrated in the preforaminal or foraminal zone, called the “second window of McCulloch” [3,4]. If the fragment is not fully migrated into McNab’s hidden zone, access to it may become very difficult, if not impossible [5]. Therefore, case selection is crucial to the success rate of the translaminar approach.

Fractures of the pars interarticularis presented an issue of the initial approach, as described by Di Lorenzo [3]. However, several authors [3,5] suggest that they can be avoided if the fenestration is more medial and oval-shaped in the caudocranial direction. Reulen’s criteria imply that the width of the lamina decreases in a cranial to caudal direction, whereas the width of the isthmus increases [3]. This should be considered when approaching the target lamina.

Patients with bony abnormalities such as foraminal spondylosis are contraindicated [4].

Initially, the approach was criticized for the supposed inability to perform a microdiscectomy through the small window. However, the studies show that it is feasible, but one should bear in mind the tight intervertebral space at the L5-S1 level, which may limit the total microdiscectomy [4].

Dural tears may occur with this approach because of the minimalistic exposure or simply because of the very thin dura that is tightly adherent to the disc fragments [6]. However, a simple management strategy is used in several studies - a patch is glued over the durotomy [5]. The narrow surgical corridor may become a potential disadvantage if the need to suture the dura occurs - this may necessitate further drilling and bony extension of the approach [6].

Postoperative results show that the translaminar approach is noninferior to the standard interlaminar approach and superior to the endoscopic transforaminal approach. Son et al. presented a retrospective study of 38 patients comparing traditional interlaminotomy to the translaminar approach - the translaminar approach group showed better ODI and VAS scores and a significantly greater recovery of total lumbar lordotic angle [7]. Schulz et al. compared traditional interlaminotomy to the translaminar approach and the endoscopic transforaminal approach - they concluded that the only statistically significant difference between the interlaminotomy and the translaminar approach is the decreased operative time in the latter, while they are both superior to the endoscopic approach in terms of clinical outcome [8].

## Conclusions

The microscopic translaminar approach is a viable alternative to standard interlaminotomy for cranially and laterally migrated LDH and access to McNab’s hidden zone. This approach is minimally invasive and allows for sparing of the bony structures - it is also noninferior in terms of patient recovery. It is cost-effective and applicable in countries where endoscopic and exoscopic spinal surgery is not widely used. However, careful preoperative planning is necessary to optimize proper patient selection and ensure the best success rate possible.
